# Circulating Procollagen Type III N-Terminal Peptide and Physical Function in the Long Life Family Study

**DOI:** 10.1093/geroni/igaa057.804

**Published:** 2020-12-16

**Authors:** Adam Santanasto, Ryan Cvejkus, Mary Wojczynski, Nicole Schupf, Megan Marron, Kaare Christensen, Bharat Thyagarajan, Joseph Zmuda

**Affiliations:** 1 University of Pittsburgh, PITTSBURGH, Pennsylvania, United States; 2 University of Pittsburgh, Pittsburgh, Pennsylvania, United States; 3 Washington University School of Medicine, St Louis, Missouri, United States; 4 Columbia University Medical Center, New York, New York, United States; 5 University of Southern Denmark, Odense C, Syddanmark, Denmark; 6 University of Minnesota, Minneapolis, Minnesota, United States

## Abstract

Circulating levels of procollagen type III N-terminal peptide (P3NP) may reflect increased fibrosis of skeletal muscle and other tissues with aging. In the current study, we tested if P3NP levels were associated with baseline and 7-year change in physical function among adults aged 39-104. Participants (n=400) were from the Long Life Family Study, a study of exceptional familial longevity. Plasma P3NP concentration was measured using a sandwich enzyme-linked immunosorbent assay (inter-assay CVs <3%). At baseline and 7-year follow-up visits, physical function was measured using the Short Physical Performance Battery (score 0-12), which consists of gait speed, balance, and chair-rise tests. Grip strength was measured using a handheld dynamometer. The association between log-transformed P3NP and physical function was examined using Generalized Estimating Equations adjusted for familial relatedness, age, sex, height, weight, lifestyle characteristics, chronic disease prevalence and inflammatory cytokines. Participants were aged 73.1 ± 15.2 years, 54% female, had a BMI of 26.6 ± 4.3 kg/m2, and a gait speed of 1.0 ± 0.3 m/s. One standard deviation higher P3NP concentration was related to worse baseline SPPB score (β=-0.9points), gait speed (β=-0.05m/s), chair-rise time (β=8.34seconds), and grip strength (β=-2.0kg; all p<0.001). Higher P3NP concentration was also associated with greater declines in gait speed (β=-1.41, p<.001) and chair-rise performance (β=0.41, p<.001). Plasma P3NP concentration may be a strong, novel biomarker of current and physical function changes with aging. Future research is needed to extend our findings to a larger population, and determine the mechanisms underlying these associations.

